# TorchIO: A Python library for efficient loading, preprocessing, augmentation and patch-based sampling of medical images in deep learning

**DOI:** 10.1016/j.cmpb.2021.106236

**Published:** 2021-09

**Authors:** Fernando Pérez-García, Rachel Sparks, Sébastien Ourselin

**Affiliations:** a Department of Medical Physics and Biomedical Engineering, University College London, UK; b Wellcome / EPSRC Centre for Interventional and Surgical Sciences (WEISS), University College London, UK; c School of Biomedical Engineering & Imaging Sciences (BMEIS), King’s College London, UK

**Keywords:** Medical image computing, Deep learning, Data augmentation, Preprocessing

## Abstract

**Background and objective:**

Processing of medical images such as MRI or CT presents different challenges compared to RGB images typically used in computer vision. These include a lack of labels for large datasets, high computational costs, and the need of metadata to describe the physical properties of voxels. Data augmentation is used to artificially increase the size of the training datasets. Training with image subvolumes or patches decreases the need for computational power. Spatial metadata needs to be carefully taken into account in order to ensure a correct alignment and orientation of volumes.

**Methods:**

We present TorchIO, an open-source Python library to enable efficient loading, preprocessing, augmentation and patch-based sampling of medical images for deep learning. TorchIO follows the style of PyTorch and integrates standard medical image processing libraries to efficiently process images during training of neural networks. TorchIO transforms can be easily composed, reproduced, traced and extended. Most transforms can be inverted, making the library suitable for test-time augmentation and estimation of aleatoric uncertainty in the context of segmentation. We provide multiple generic preprocessing and augmentation operations as well as simulation of MRI-specific artifacts.

**Results:**

Source code, comprehensive tutorials and extensive documentation for TorchIO can be found at http://torchio.rtfd.io/. The package can be installed from the Python Package Index (PyPI) running pip install torchio. It includes a command-line interface which allows users to apply transforms to image files without using Python. Additionally, we provide a graphical user interface within a TorchIO extension in 3D Slicer to visualize the effects of transforms.

**Conclusion:**

TorchIO was developed to help researchers standardize medical image processing pipelines and allow them to focus on the deep learning experiments. It encourages good open-science practices, as it supports experiment reproducibility and is version-controlled so that the software can be cited precisely. Due to its modularity, the library is compatible with other frameworks for deep learning with medical images.

## Highlights

• Open-source Python library for preprocessing, augmentation and sampling of medical images for deep learning.
• Support for 2D, 3D and 4D images such as X-ray, histopathology, CT, ultrasound and diffusion MRI.
• Modular design inspired by the deep learning framework PyTorch.
• Focus on reproducibility and traceability to encourage open-science practices.
• Compatible with related frameworks for medical image processing with deep learning.

## 1 Introduction

Recently, deep learning has become a ubiquitous research approach for solving image understanding and analysis problems. Convolutional neural networks (CNNs) have become the state of the art for many medical imaging tasks including segmentation [1], classification [2], reconstruction [3] and registration [4]. Many of the network architectures and techniques have been adopted from computer vision.

Compared to 2D red-green-blue (RGB) images typically used in computer vision, processing of medical images such as MRI, ultrasound (US) or CT presents different challenges. These include a lack of labels for large datasets, high computational costs (as the data is typically volumetric), and the use of metadata to describe the physical size and position of voxels.

Open-source frameworks for training CNNs with medical images have been built on top of TensorFlow [5], [6], [7]. Recently, the popularity of PyTorch [8] has increased among researchers due to its improved usability compared to TensorFlow [9], driving the need for open-source tools compatible with PyTorch. To reduce duplication of effort among research groups, improve experimental reproducibility and encourage open-science practices, we have developed TorchIO: an open-source Python library for efficient loading, preprocessing, augmentation, and patch-based sampling of medical images designed to be integrated into deep learning workflows.

TorchIO is a compact and modular library that can be seamlessly used alongside higher-level deep learning frameworks for medical imaging, such as the Medical Open Network for AI (MONAI). It removes the need for researchers to code their own preprocessing pipelines from scratch, which might be error-prone due to the complexity of medical image representations. Instead, it allows researchers to focus on their experiments, supporting experiment reproducibility and traceability of their work, and standardization of the methods used to process medical images for deep learning.

### 1.1 Motivation

The nature of medical images makes it difficult to rely on a typical computer-vision pipeline for neural network training. In Section 1.1.1, we describe challenges related to medical images that need to be overcome when designing deep learning workflows. In Section 1.1.2, we justify the choice of PyTorch as the main deep learning framework dependency of TorchIO.

#### 1.1.1 Challenges in medical image processing for deep learning

In practice, multiple challenges must be addressed when developing deep learning algorithms for medical images: 1) handling metadata related to physical position and size, 2) lack of large labeled datasets, 3) high computational costs due to data multidimensionality and 4) lack of consensus for best normalization practices. These challenges are very common in medical imaging and require certain features that may not be implemented in more general-purpose image processing frameworks such as Albumentations [10] or TorchVision [8].

##### Metadata

In computer vision, picture elements, or *pixels*, which are assumed to be square, have a spatial relationship that comprises proximity and depth according to both the arrangement of objects in the scene and camera placement. In comparison, medical images are reconstructed such that the location of volume elements, or cuboid-shaped *voxels*, encodes a meaningful 3D spatial relationship. In simple terms, for 2D natural images, pixel vicinity does not necessarily indicate spatial correspondence, while for medical images spatial correspondence between nearby voxels can often be assumed.

Metadata, which encodes the physical size, spacing, and orientation of voxels, determines spatial relationships between voxels [11]. This information can provide meaningful context when performing medical image processing, and is often implicitly or explicitly used in medical imaging software. Furthermore, metadata is often used to determine correspondence between images as well as voxels within an image. For example, registration algorithms for medical images typically work with physical coordinates rather than voxel indices.

**Fig. 1:**
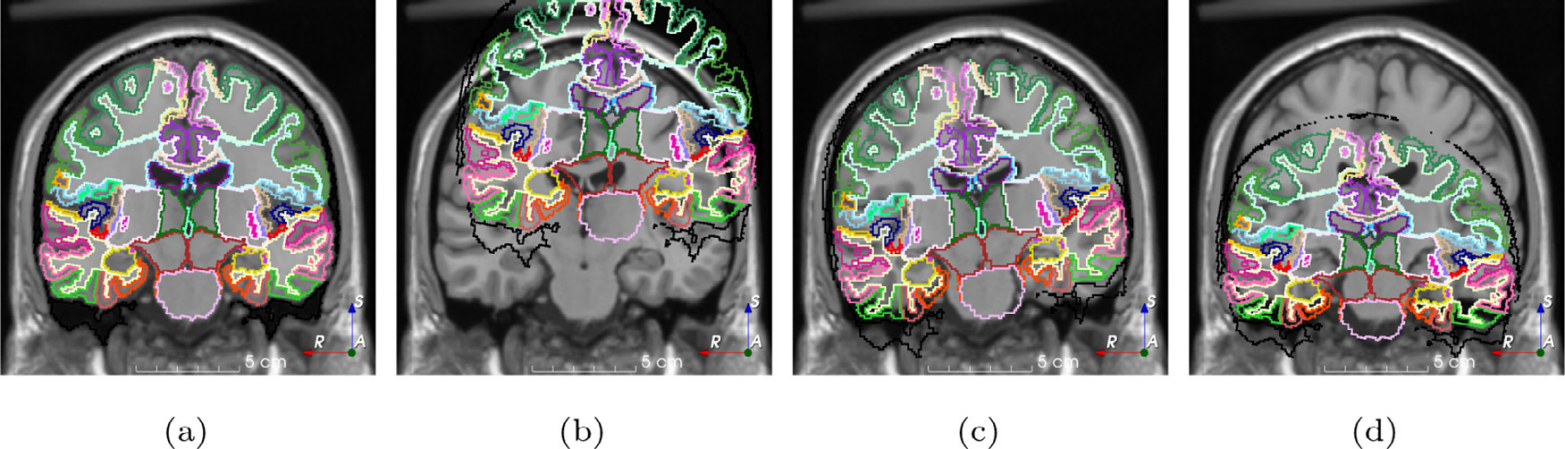
Demonstration of the importance of spatial metadata in medical image processing. The size of both the MRI and the segmentation is 181×181. When spatial metadata is taken into account (a), images are correctly superimposed (only the borders of each region are shown for clarity purposes). Images are incorrectly superimposed if (b) origin, (c) orientation or (d) spacing are ignored.

Fig. 1 shows the superposition of an MRI and a corresponding brain parcellation [12] with the same size (181×181) but different origin, spacing and orientation. A native user would assume that, given that the superimposition looks correct and both images have the same size, they are ready for training. However, the visualization is correct only because 3D Slicer [13], the software used for visualization, is aware of the spatial metadata of the images. As CNNs generally do not take spatial metadata into account, training using these images without preprocessing would lead to poor results.

Medical images are typically stored in specialized formats such as Data Imaging and Communications in Medicine (DICOM) or Neuroimaging Informatics Technology Initiative (NIfTI) [11], and commonly read and processed by medical imaging frameworks such as SimpleITK [14] or NiBabel [15].

*Limited training data.* Deep learning methods typically require large amounts of annotated data, which are often scarce in clinical scenarios due to concerns over patient privacy, the financial and time burden associated with collecting data as part of a clinical trial, and the need for annotations from highly-trained and experienced raters. Data augmentation techniques can be used to increase the size of the training dataset artificially by applying different transformations to each training instance while preserving the relationship to annotations.

Data augmentation performed in computer vision typically aims to simulate variations in camera properties, field of view (FOV), or perspective. Traditional data augmentation operations applied in computer vision include geometrical transforms such as random rotation or zoom, color-space transforms such as random channel swapping or kernel filtering such as random Gaussian blurring. Data augmentation is usually performed on the fly, i.e., every time an image is loaded from disk during training.

Several computer vision libraries supporting data augmentation have appeared recently, such as Albumentations [10], or imgaug [16]. PyTorch also includes some computer vision transforms, mostly implemented as Pillow wrappers [17]. However, none of these libraries support reading or transformations for 3D images. Furthermore, medical images are almost always grayscale, therefore color-space transforms are not applicable. Additionally, cropping and scaling are more challenging to apply to medical images without affecting the spatial relationships of the data. Metadata should usually be considered when applying these transformations to medical images.

In medical imaging, the purpose of data augmentation is designed to simulate anatomical variations and scanner artifacts. Anatomical variation and sample position can be simulated using spatial transforms such as elastic deformation, lateral flipping, or affine transformations. Some artifacts are unique to specific medical image modalities. For example, ghosting artifacts will be present in MRI if the patient moves during acquisition, and metallic implants often produce streak artifacts in CT. Simulation of these artifacts can be useful when performing augmentation on medical images.

*Computational costs.* The number of pixels in 2D images used in deep learning is rarely larger than one million. For example, the input size of several popular image classification models is 224×224×3=150528 pixels (588 KiB if 32 bits per pixel are used). In contrast, 3D medical images often contain hundreds of millions of voxels, and downsampling might not be acceptable when small details should be preserved. For example, the size of a high-resolution lung CT-scan used for quantifying chronic obstructive pulmonary disease (COPD) damage in a research setting, with spacing 0.66×0.66×0.30 mm, is 512×512×1069=280231936 voxels (1.04 GiB if 32 bits per voxel are used).

In computer vision applications, images used for training are grouped in batches whose size is often in the order of hundreds [18] or even thousands [19] of training instances, depending on the available graphics processing unit (GPU) memory. In medical image applications, batches rarely contain more than one [1] or two [20] training instances due to their larger memory footprint compared to natural images. This reduces the utility of techniques such as batch normalization, which rely on batches being large enough to estimate dataset variance appropriately [21]. Moreover, large image size and small batches result in longer training time, hindering the experimental cycle that is necessary for hyperparameter optimization. In cases where GPU memory is limited and the network architecture is large, it is possible that not even the entirety of a single volume can be processed during a training iteration. To overcome this challenge, it is common in medical imaging to train using subsets of the image, or image *patches*, randomly extracted from the volumes.

Networks can be trained with 2D slices extracted from 3D volumes, aggregating the inference results to generate a 3D volume [22]. This can be seen as a specific case of patch-based training, where the size of the patches along a dimension is one. Other methods extract volumetric patches for training, that are often cubes, if the voxel spacing is isotropic [23], or cuboids adapted to the anisotropic spacing of the training images [24].

*Transfer learning and normalization.* One can pre-train a network on a large dataset of natural images such as ImageNet [25], which contains more than 14 million labeled images, and fine-tune on a custom, much smaller target dataset. This is a typical use of transfer learning in computer vision [26]. The literature has reported mixed results using transfer learning to apply models pretrained on natural images to medical images [27], [28].

In computer vision, best practice is to normalize each training instance before training, using statistics computed from the whole training dataset [18]. Preprocessing of medical images is often performed on a per-image basis, and best practice is to take into account the bimodal nature of medical images (i.e., that an image has a background and a foreground).

Medical image voxel intensity values can be encoded with different data types and intensity ranges, and the meaning of a specific value can vary between different modalities, sequence acquisitions, or scanners. Therefore, intensity normalization methods for medical images often involve more complex parameterization of intensities than those used for natural images [29].

##### 1.1.2 Deep learning frameworks

There are currently two major generic deep learning frameworks: TensorFlow [5] and PyTorch [8], primarily maintained by Google and Facebook, respectively. Although TensorFlow has traditionally been the primary choice for both research and industry, PyTorch has recently seen a substantial increase in popularity, especially among the research community [9].

PyTorch is often preferred by the research community as it is *pythonic*, i.e., its design, usage, and application programming interfaceAPI follow the conventions of plain Python. Moreover, the API for tensor operations follows a similar paradigm to the one for NumPy multidimensional arrays, which is the primary array programming library for the Python language [30]. In contrast, for TensorFlow, researchers need to become familiar with new design elements such as sessions, placeholders, feed dictionaries, gradient tapes and static graphs. In PyTorch, objects are standard Python classes and variables, and a dynamic graph makes debugging intuitive and familiar to anyone already using Python. These differences have decreased with the recent release of TensorFlow 2, whose eager mode makes usage reminiscent of Python.

TorchIO was designed to be in the style of PyTorch and uses several of its tools to reduce the barrier to learning how to use TorchIO for those researchers already familiar with PyTorch.

#### 1.2 Related work

NiftyNet [7] and the Deep Learning Toolkit (DLTK) [6] are deep learning frameworks designed explicitly for medical image processing using the TensorFlow 1 platform. Both of them are no longer being actively maintained. They provide implementations of some popular network architectures such as U-Net [1], and can be used to train 3D CNNs for different tasks. For example, NiftyNet was used to train a 3D residual network for brain parcellation [23], and DLTK was used to perform multi-organ segmentation on CT and MRI [31].

The medicaltorch library [32] closely follows the PyTorch design, and provides some functionalities for preprocessing, augmentation and training of medical images. However, it does not leverage the power of specialized medical image processing libraries, such as SimpleITK [14], to process volumetric images.

Similar to DLTK, this library has not seen much activity since 2018.

The batchgenerators library [33], used within the popular medical segmentation framework nn-UNet [34], includes custom dataset and data loader classes for multithreaded loading of 3D medical images, implemented before data loaders were available in PyTorch. In the usage examples from GitHub, preprocessing is applied to the whole dataset before training. Then, spatial data augmentation is performed at the volume level, from which one patch is extracted and intensity augmentation is performed at the patch level. In this approach, only one patch is extracted per volume, diminishing the efficiency of training pipelines. Transforms in batchgenerators are mostly implemented using NumPy [30] and SciPy [35].

More recently, a few PyTorch-based libraries for deep learning and medical images have appeared. There are two other libraries, developed in parallel to TorchIO, focused on data preprocessing and augmentation. Rising^1^ is a library for data augmentation entirely written in PyTorch, which allows for gradients to be propagated through the transformations and perform all computations on the GPU. However, this means specialized medical imaging libraries such as SimpleITK cannot be used. pymia [36] provides features for data handling (loading, preprocessing, sampling) and evaluation. It is compatible with TorchIO transforms, which are typically leveraged for data augmentation, as their data handling is more focused on preprocessing. pymia can be easily integrated into either PyTorch or TensorFlow pipelines. It was recently used to assess the suitability of evaluation metrics for medical image segmentation [37].

MONAI [38] and Eisen [39] are PyTorch-based frameworks for deep learning workflows with medical images. Similar to NiftyNet and DLTK, they include implementation of network architectures, transforms, and higher-level features to perform training and inference. For example, MONAI was recently used for brain segmentation on fetal MRI [40]. As these packages are solving a large problem, i.e., that of workflow in deep learning for medical images, they do not contain all of the data augmentation transforms present in TorchIO. However, it is important to note that an end user does not need to select only one open-source package, as TorchIO transforms are compatible with both Eisen and MONAI.

TorchIO is a library that specializes in preprocessing and augmentation using PyTorch, focusing on ease of use for researchers. This is achieved by providing a PyTorch-like API, comprehensive documentation with many usage examples, and tutorials showcasing different features, and by actively addressing feature requests and bug reports from the many users that have already adopted TorchIO. This is in contrast with other modern libraries released after TorchIO such as MONAI, which aims to deliver a larger umbrella of functionalities including federated learning or active learning, but may have slower development and deployment.

## 2 Methods

We developed TorchIO, a Python library that focuses on data loading and augmentation of medical images in the context of deep learning.

TorchIO is a unified library to load and augment data that makes explicit use of medical image properties, and is flexible enough to be used for different loading workflows. It can accelerate research by avoiding the need to code a processing pipeline for medical images from scratch.

In contrast with Eisen or MONAI, we do not implement network architectures, loss functions or training workflows. This is to limit the scope of the library and to enforce modularity between training of neural networks and preprocessing and data augmentation.

Following the PyTorch philosophy [8], we designed TorchIO with an emphasis on simplicity and usability while reusing PyTorch classes and infrastructure where possible. Note that, although we designed TorchIO following PyTorch style, the library could also be used with other deep learning platforms such as TensorFlow or Keras [41].

TorchIO makes use of open-source medical imaging software platforms. Packages were selected to reduce the number of required external dependencies and the need to re-implement basic medical imaging processing operations (image loading, resampling, etc.).

**Fig. 2:**
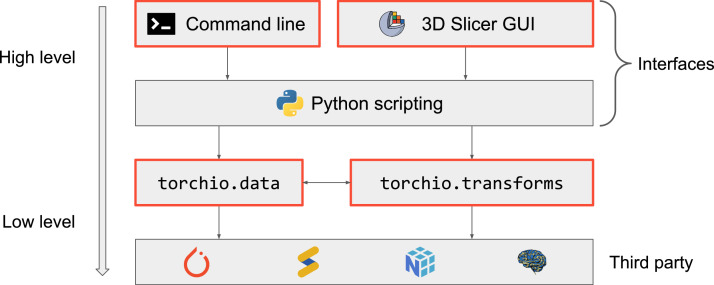
General diagram of TorchIO, its dependencies and its interfaces. Boxes with a red border () represent elements implemented in TorchIO. Logos indicate lower-level Python libraries used by TorchIO. : NiBabel [15]; : SimpleITK [14]; : NumPy [30]; : PyTorch [8].

TorchIO features are divided into two categories: data structures and input/output (torchio.data), and transforms for preprocessing and augmentation (torchio.transforms). Fig. 2 represents a diagram of the codebase and the different interfaces to the library.

### 2.1 Data

#### 2.1.1 Input/Output

TorchIO uses the medical imaging libraries NiBabel and SimpleITK to read and write images. Dependency on both is necessary to ensure broad support of image formats. For instance, NiBabel does not support reading Portable Network Graphics (PNG) files, while SimpleITK does not support some neuroimaging-specific formats.

TorchIO supports up to 4D images, i.e., 2D or 3D single-channel or multi-channel data such as X-rays, RGB histological slides, microscopy stacks, multispectral images, CT-scans, functional MRI (fMRI) and diffusion MRI (dMRI).

#### 2.1.2 Data structures

*Image.* The Image class, representing one medical image, stores a 4D tensor, whose voxels encode, e.g., signal intensity or segmentation labels, and the corresponding affine transform, typically a rigid (Euclidean) transform, to convert voxel indices to world coordinates in millimeters. Arbitrary fields such as acquisition parameters may also be stored.

Subclasses are used to indicate specific types of images, such as ScalarImage and LabelMap, which are used to store, e.g., CT scans and segmentations, respectively.

An instance of Image can be created using a filepath, a PyTorch tensor, or a NumPy array. This class uses lazy loading, i.e., the data is not loaded from disk at instantiation time. Instead, the data is only loaded when needed for an operation (e.g., if a transform is applied to the image).

**Fig. 3:**
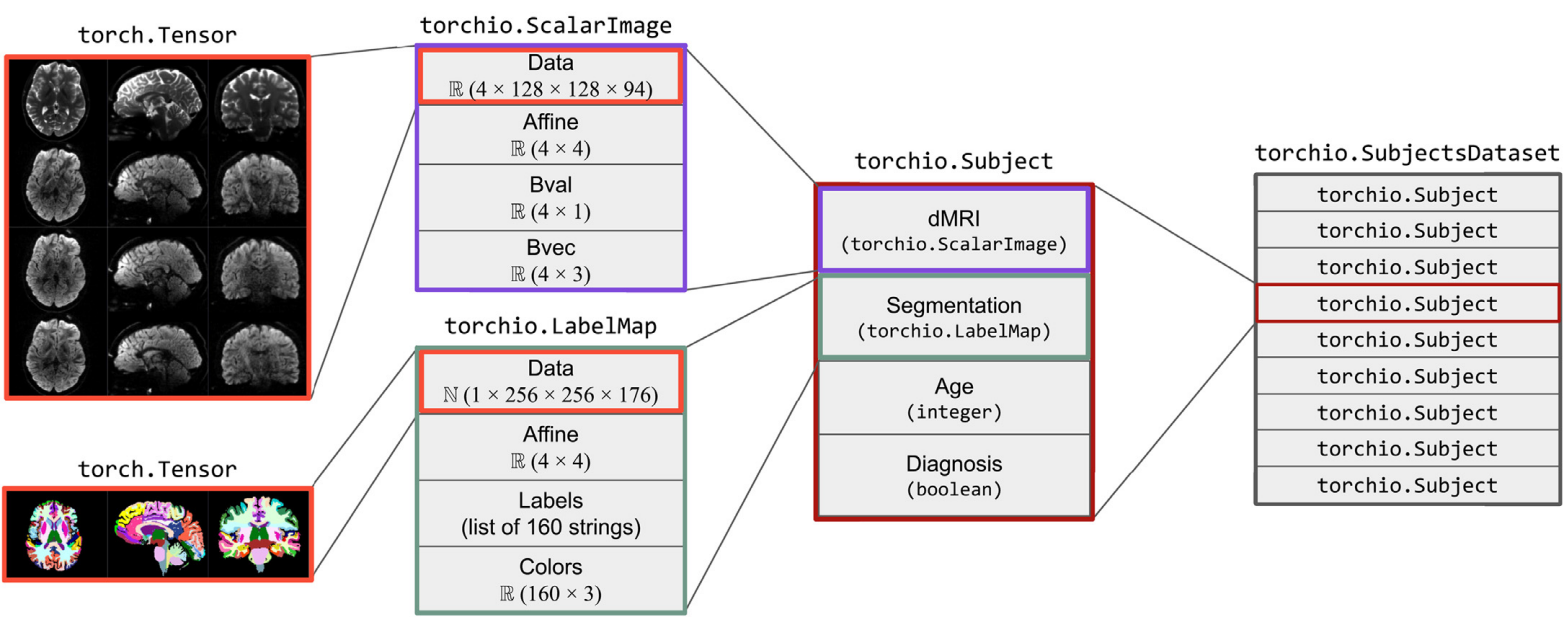
Usage example of ScalarImage, LabelMap, Subject and SubjectsDataset. The images store a 4D dMRI and a brain parcellation, and other related metadata.

Fig. 3 shows two instances of Image. The instance of ScalarImage contains a 4D tensor representing a dMRI, which contains four 3D volumes (one per gradient direction), and the associated affine matrix. Additionally, it stores the strength and direction for each of the four gradients. The instance of LabelMap contains a brain parcellation of the same subject, the associated affine matrix, and the name and color of each brain structure.

*Subject.* The Subject class stores instances of Image associated to a subject, e.g., a human or a mouse. As in the Image class, Subject can store arbitrary fields such as age, diagnosis or ethnicity.

*Subjects dataset*. The SubjectsDataset inherits from the PyTorch Dataset. It contains the list of subjects and optionally a transform to be applied to each subject after loading. When SubjectsDataset is queried for a specific subject, the corresponding set of images are loaded, a transform is applied to the images and the instance of Subject is returned.

**Fig. 4:**
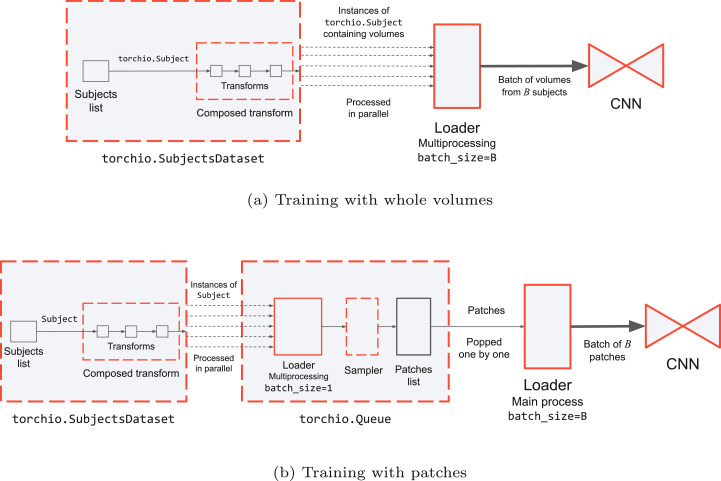
Diagram of data pipelines for training with whole volumes (top) and patches (bottom). Boxes with a red border represent PyTorch classes () or TorchIO classes that inherit from PyTorch classes ().

For parallel loading, a PyTorch DataLoader may be used. This loader spawns multiple processes, each of which contains a shallow copy of the SubjectsDataset. Each copy is queried for a different subject, therefore loading and transforming is applied to different subjects in parallel on the central processing unit (CPU) (Fig. 4a).

An example of subclassing SubjectsDataset is torchio.datasets.IXI, which may be used to download the Information eXtraction from Images (IXI) dataset.^2^

#### 2.1.3 Patch-based training

Memory limitations often require training and inference steps to be performed using image subvolumes or *patches* instead of the whole volumes, as explained in Section 1.1.1.3. In this section, we describe how TorchIO implements patch-based training via image sampling and queueing.

*Samplers.* A sampler takes as input an instance of Subject and returns a version of it whose images have a reduced FOV, i.e., the new images are subvolumes, also called windows or *patches*. For this, a PatchSampler may be used.

Different criteria may be used to select the center voxel of each output patch. A UniformSampler selects a voxel as the center at random with all voxels having an equal probability of being selected. A WeightedSampler selects the patch center according to a probability distribution image defined over all voxels, which is passed as input to the sampler.

At testing time, images are sampled such that a dense inference can be performed on the input volume. A GridSampler can be used to sample patches such that the center voxel is selected using a set stride. In this way, sampling over the entire volume is ensured. The potentially-overlapping inferred patches can be passed to a GridAggregator that builds the resulting volume patch by patch (or batch by batch).

*Queue.* A training iteration (i.e., forward and backward pass) performed on a GPU is usually faster than loading, preprocessing, augmenting, and cropping a volume on a CPU. Most preprocessing operations could be performed using a GPU, but these devices are typically reserved for training the CNN so that the batch size and input tensor can be as large as possible. Therefore, it is beneficial to prepare (i.e., load, preprocess and augment) the volumes using multiprocessing CPU techniques in parallel with the forward-backward passes of a training iteration.

Once a volume is appropriately prepared, it is computationally beneficial to sample multiple patches from a volume rather than having to prepare the same volume each time a patch needs to be extracted. The sampled patches are then stored in a buffer or *queue* until the next training iteration, at which point they are loaded onto the GPU to perform an optimization iteration. For this, TorchIO provides the Queue class, which inherits from the PyTorch Dataset (Fig. 4b). In this queueing system, samplers behave as generators that yield patches from volumes contained in the SubjectsDataset.

The end of a training epoch is defined as the moment after which patches from all subjects have been used for training. At the beginning of each training epoch, the subjects list in the SubjectsDataset is shuffled, as is typically done in machine learning pipelines to increase variance of training instances during model optimization. A PyTorch loader begins by shallow-copying the dataset to each subprocess. Each worker subprocess loads and applies image transforms to the volumes in parallel. A patches list is filled with patches extracted by the sampler, and the queue is shuffled once it has reached a specified maximum length so that batches are composed of patches from different subjects. The internal data loader continues querying the SubjectsDataset using multiprocessing. The patches list, when emptied, is refilled with new patches. A second data loader, external to the queue, may be used to collate batches of patches stored in the queue, which are passed to the neural network.

### 2.2 Transforms

**Fig. 5:**
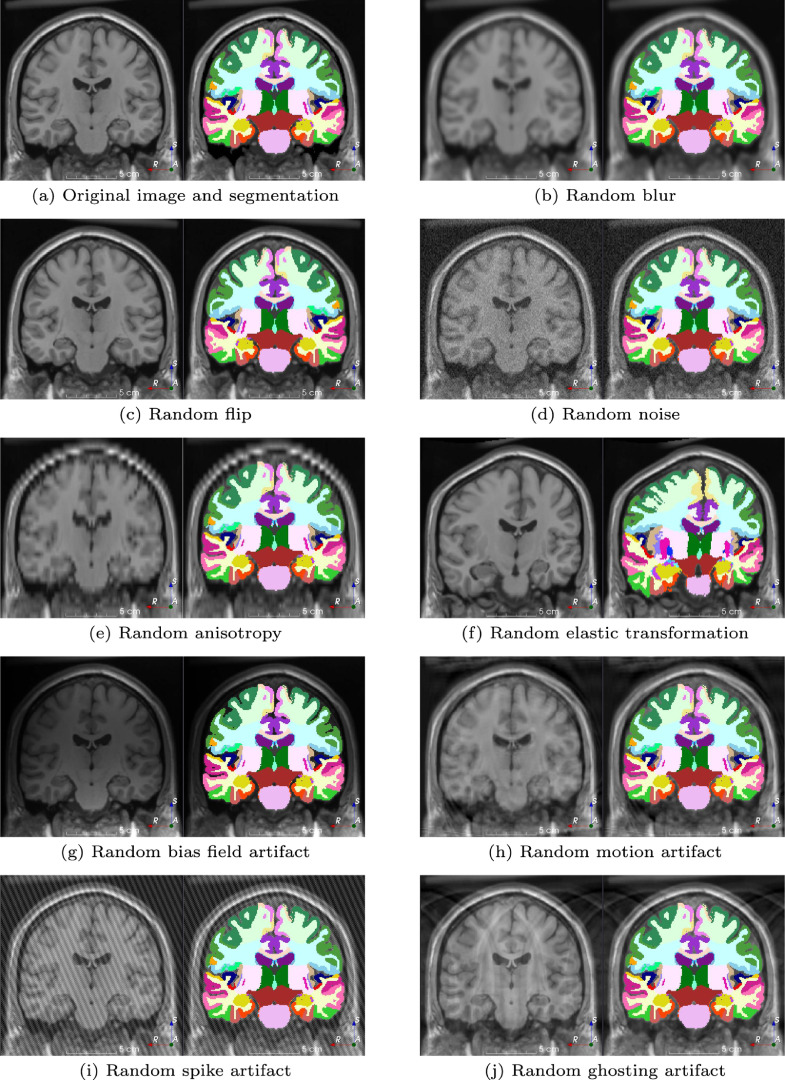
A selection of data augmentation techniques available in TorchIO v0.18.0. Each example is presented as a pair of images composed of the transformed image and a corresponding transformed label map. Note that all screenshots are from a 2D coronal slice of the transformed 3D images. The MRI corresponds to the Montreal Neurological Institute (MNI) Colin 27 average brain [49], which can be downloaded using torchio.datasets.Colin27. Label maps were generated using an automated brain parcellation algorithm [12].

The transforms API was designed to be similar to the PyTorch

torchvision.transforms module. TorchIO includes augmentations such as random affine transformation (Fig. 5e) or random blur (Fig. 5b), but they are implemented using medical imaging libraries [14], [15] to take into account specific properties of medical images, namely their size, resolution, location, and orientation (see Section 1.1.1.1). Table 1 shows transforms implemented in TorchIO v0.18.0 and their main corresponding library dependencies.

**Table 1:** Transforms included in TorchIO v0.18.0. Logos indicate the main library used to process the images. : NiBabel [15]; : SimpleITK [14]; : NumPy [30]; : PyTorch [8], [43], [45].

Transforms are designed to be flexible regarding input and output types. Following a duck typing approach, they can take as input PyTorch tensors, SimpleITK images, NumPy arrays, Pillow images, Python dictionaries, and instances of Subject and Image, and will return an output of the same type.

TorchIO transforms can be classified into either spatial and intensity transforms, or preprocessing and augmentation transforms (Table 1). All are subclasses of the Transform base class. Spatial transforms and intensity transforms are related to the SpatialTransform and IntensityTransform classes, respectively. Transforms whose parameters are randomly chosen are subclasses of RandomTransform.

Instances of SpatialTransform typically modify the image bounds or spacing, and often need to resample the image using interpolation. They are applied to all image types. Instances of IntensityTransform do not modify the position of voxels, only their values, and they are only applied to instances of ScalarImage. For example, if a RandomNoise transform (which is a subclass of IntensityTransform) receives as input a Subject with a ScalarImage representing a CT scan and a LabelMap representing a segmentation, it will add noise to only the CT scan. On the other hand, if a RandomAffine transform (which is a subclass of SpatialTransform) receives the same input, the same affine transformation will be applied to both images, with nearest-neighbor interpolation always used to interpolate LabelMap objects.

#### 2.2.1 Preprocessing

Preprocessing transforms are necessary to ensure spatial and intensity uniformity of training instances.

Spatial preprocessing is important as CNNs do not generally take into account metadata related to medical images (see Section 1.1.1.1), therefore it is necessary to ensure that voxels across images have similar spatial location and relationships before training. Spatial preprocessing transforms typically used in medical imaging include resampling (e.g., to make voxel spacing isotropic for all training samples) and reorientation (e.g., to orient all training samples in the same way). For example, the Resample transform can be used to fix the issue presented in Fig. 1.

Intensity normalization is generally beneficial for optimization of neural networks. TorchIO provides intensity normalization techniques including min-max scaling or standardization,^3^ which are computed using pure PyTorch. A binary image, such as a mask representing the foreground or structures of interest, can be used to define the set of voxels to be taken into account when computing statistics for intensity normalization. We also provide a method for MRI histogram standardization [48], computed using NumPy, which may be used to overcome the differences in intensity distributions between images acquired using different scanners or sequences.

#### 2.2.2 Augmentation

TorchIO includes spatial augmentation transforms such as random flipping using PyTorch and random affine and elastic deformation transforms using SimpleITK. Intensity augmentation transforms include random Gaussian blur using a SimpleITK filter (Fig. 5b) and addition of random Gaussian noise using pure PyTorch (Fig. 5d). All augmentation transforms are subclasses of RandomTransform.

Although current domain-specific data augmentation transforms available in TorchIO are mostly related to MRI, we encourage users to contribute physics-based data augmentation techniques for US or CT [50].

We provide several MRI-specific augmentation transforms related to k-space, which are described below. An MR image is usually reconstructed as the magnitude of the inverse Fourier transform of the k-space signal, which is populated with the signals generated by the sample as a response to a radio-frequency electromagnetic pulse. These signals are modulated using coils that create gradients of the magnetic field inside the scanner. Artifacts are created by using k-space transforms to perturb the Fourier space and generate corresponding intensity artifacts in image space. The forward and inverse Fourier transforms are computed using the Fast Fourier Transform (FFT) algorithm implemented in NumPy.

*Random*k*-space spike artifact.* Gradients applied at a very high duty cycle may produce bad data points, or noise spikes, in k-space [51]. These points in k-space generate a spike artifact, also known as Herringbone, crisscross or corduroy artifact, which manifests as uniformly-separated stripes in image space, as shown in Fig. 5i. This type of data augmentation has recently been used to estimate uncertainty through a heteroscedastic noise model [44].

*Random*k*-space motion artifact*. The k-space is often populated line by line, and the sample in the scanner is assumed to remain static. If a patient moves during the MRI acquisition, motion artifacts will appear in the reconstructed image. We implemented a method to simulate random motion artifacts (Fig. 5h) that has been used successfully for data augmentation to model uncertainty and improve segmentation [42].

*Random*k*-space ghosting artifact*. Organs motion such as respiration or cardiac pulsation may generate ghosting artifacts along the phase-encoding direction [51] (see Fig. 5j). We simulate this phenomenon by removing every nth plane of the k-space along one direction to generate n ghosts along that dimension, while keeping the center of k-space intact.

*Random bias field artifact.* Inhomogeneity of the static magnetic field in the MRI scanner produces intensity artifacts of very low spatial frequency along the entirety of the image. These artifacts can be simulated using polynomial basis functions [52], as shown in Fig. 5g.

#### 2.2.3 Composability

**Fig. 6:**
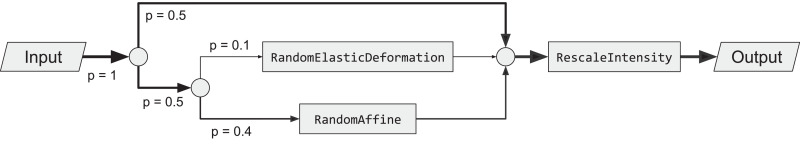
Graph representation of the composed transform described in Section 2.2.3.



All transforms can be composed in a linear fashion, as in the PyTorch torchvision library, or building a directed acyclic graphDAG using the OneOf transform (as in [10]). For example, a user might want to apply a random spatial augmentation transform to 50% of the samples using either an affine or an elastic transform, but they want the affine transform to be applied to 80% of the augmented images, as the execution time is faster. Then, they might want to rescale the volume intensity for all images to be between 0 and 1. Fig. 6 shows a graph representing the transform composition. This transform composition can be implemented with just three statements:

Compose and OneOf are implemented as TorchIO transforms.

#### 2.2.4 Extensibility

The Lambda transform can be passed an arbitrary callable object, which allows the user to augment the library with custom transforms without having a deep understanding of the underlying code.

Additionally, more complex transforms can be developed. For example, we implemented a TorchIO transform to simulate brain resection cavities from preoperative MR images within a self-supervised learning pipeline [53]. The RandomLabelsToImage transform may be used to simulate an image from a tissue segmentation. It can be composed with RandomAnisotropy to train neural networks agnostic to image contrast and resolution [46], [47], [54].

#### 2.2.5 Reproducibility and traceability

To promote open science principles, we designed TorchIO to support experiment reproducibility and traceability.

All transforms support receiving Python primitives as arguments, which makes TorchIO suitable to be used with a configuration file associated to a specific experiment.

A history of all applied transforms and their computed random parameters is saved in the transform output so that the path in the DAG and the parameters used can be traced and reproduced. Furthermore, the Subject class includes a method to compose the transforms history into a single transform that may be used to reproduce the exact result (Section 2.2.3).

#### 2.2.6 Invertibility

Inverting transforms is especially useful in scenarios where one needs to apply some transformation, infer a segmentation on the transformed data and then apply the inverse transformation to bring the inference into the original image space. The Subject class includes a method to invert the transformations applied. It does this by first inverting all transforms that are invertible, discarding the ones that are not. Then, it composes the invertible transforms into a single transform.

Transforms invertibility is most commonly applied to test-time augmentation [55] or estimation of aleatoric uncertainty [56] in the context of image segmentation.

## 3 Results

### 3.1 Code availability

All the code for TorchIO is available on GitHub^4^. We follow the semantic versioning system [57] to tag and release our library. Releases are published on the Zenodo data repository^5^ to allow users to cite the specific version of the package they used in their experiments. The version described in this paper is v0.18.0 [58]. Detailed API documentation is hosted on Read the Docs and comprehensive Jupyter notebook tutorials are hosted on Google Colaboratory, where users can run examples online. The library can be installed with a single line of code on Windows, macOS or Linux using the Pip Installs Packages (PIP) package manager: pip install torchio.

TorchIO has a strong community of users, with more than 900 stars on GitHub and more than 7000 Python Package Index (PyPI) downloads per month^6^ as of July 2021.

#### 3.1.1 Additional interfaces

The provided command-line interface (CLI) tool torchio-transform allows users to apply a transform to an image file without using Python. This tool can be used to visualize only the preprocessing and data augmentation pipelines and aid in experimental design for a given application. It can also be used in shell scripts to preprocess and augment datasets in cases where large storage is available and on-the-fly loading needs to be faster.

**Fig. 7:**
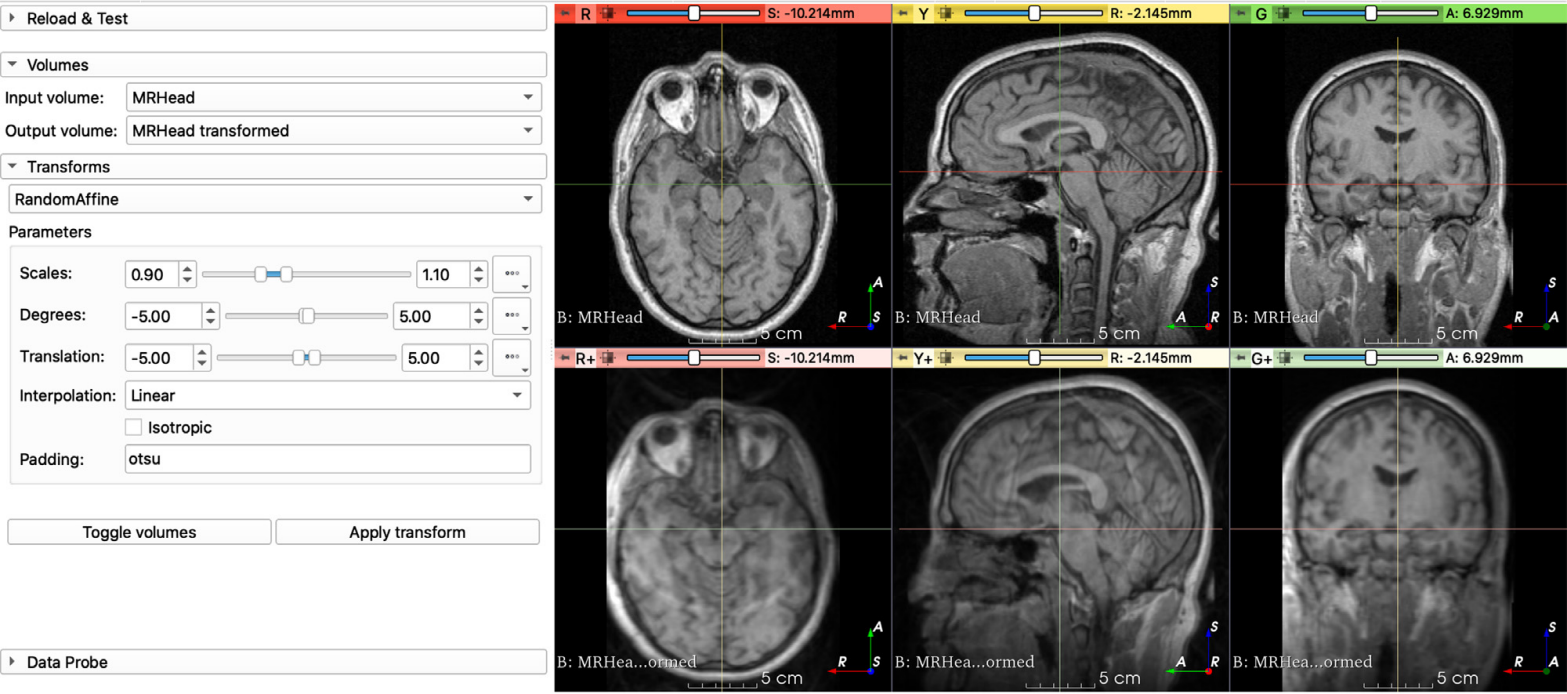
GUI for TorchIO, implemented as a 3D Slicer extension. In this example, the applied transforms are RandomBiasField, RandomGhosting, RandomMotion, RandomAffine and RandomElasticDeformation.

Additionally, we provide a graphical user interface (GUI) implemented as a Python scripted module within the *TorchIO* extension available in 3D Slicer [13]. It can be used to visualize the effect of the transforms parameters without any coding (Fig. 7). As with the CLI tool, users can experimentally assess preprocessing and data augmentation before network training to ensure the preprocessing pipeline is suitable for a given application.

### 3.2 Usage examples

In this section, we briefly describe the implementations of two medical image computing papers from the literature, pointing out the TorchIO features that could be used to replicate their experiments.

#### 3.2.1 Super-resolution and synthesis of MRI

In [54], a method is proposed to simulate high-resolution $T_{1}$-weighted MRIs from images of different modalities and resolutions.

First, brain regions are segmented on publicly available datasets of brain MRI. During training, an MRI (ScalarImage) and the corresponding segmentation (LabelMap) corresponding to a specific subject (Subject) are sampled from the training dataset (SubjectsDataset). Next, the same spatial augmentation transform is applied to both images by composing an affine transform (RandomAffine) and a nonlinear diffeomorphic transform (RandomElasticDeformation). Then, a Gaussian mixture modelGMM conditioned on the labels is sampled at each voxel location to simulate an MRI of arbitrary contrast (RandomLabelsToImage) [46]. Finally, multiple degrading phenomena are simulated on the synthetic image: variability in the coordinate frames (RandomAffine), bias field inhomogeneities (RandomBiasField), partial-volume effects due to a large slice thickness during acquisition [47] (RandomAnisotropy), registration errors (RandomAffine), and resampling artifacts (Resample).

#### 3.2.2 Adaptive sampling for segmentation of CT scans

In [59], CT scans that are too large to fit on a GPU are segmented using patch-based training with weighted sampling of patches. Discrepancies between labels and predictions are used to create error maps and patches are preferentially sampled from voxels with larger error.

During training, a CT scan (ScalarImage) and its corresponding segmentation (LabelMap) from a subject (Subject) are loaded and the same augmentation is performed to both by applying random rotations and scaling (RandomAffine). Then, voxel intensities are clipped to [−1000,1000] (RescaleIntensity) and divided by a constant factor representing the standard deviation of the dataset (can be implemented with Lambda). As the CT scans are too large to fit in the GPU, patch-based training is used (Queue). To obtain high-resolution predictions and a large receptive field simultaneously, two patches of similar size but different FOV are generated from each sampled patch: a context patch generated by downsampling the original patch (Resample) and a full-resolution patch with a smaller FOV (CropOrPad). At the end of each epoch, error maps for each subject (Subject) are computed as the difference between the labels and predictions. The error maps are used in the following epoch to sample patches with large errors more often (WeightedSampler). At inference time, a sliding window (GridSampler) is used to predict the segmentation patch by patch, and patches are aggregated to build the prediction for the whole input volume (GridAggregator).

## 4 Discussion

We have presented TorchIO, a new library to efficiently load, preprocess, augment and sample medical imaging data during the training of CNNs. It is designed in the style of the deep learning framework PyTorch to provide medical imaging specific preprocessing and data augmentation algorithms.

The main motivation for developing TorchIO as an open-source toolkit is to help researchers standardize medical image processing pipelines and allow them to focus on the deep learning experiments. It also encourages good open-science practices, as it supports experiment reproducibility and is version-controlled so that the software can be cited precisely.

The library is compatible with other higher-level deep learning frameworks for medical imaging such as MONAI. For example, users can benefit from TorchIO’s MRI transforms and patch-based sampling while using MONAI’s networks, losses, training pipelines and evaluation metrics.

The main limitation of TorchIO is that most transforms are not differentiable. The reason is that PyTorch tensors stored in TorchIO data structures must be converted to SimpleITK images or NumPy arrays within most transforms, making them not compatible with PyTorch’s automatic differentiation engine. However, compatibility between PyTorch and ITK has recently been improved, partly thanks to the appearance of the MONAI project [60]. Therefore, TorchIO might provide differentiable transforms in the future, which could be used to implement, e.g., spatial transformer networks for image registration [61]. Another limitation is that many more transforms that are MRI-specific exist than for other imaging modalities such as CT or US. This is in part due to more users working on MRI applications and requesting MRI-specific transforms. However, we welcome contributions for other modalities as well.

In the future, we will work on extending the preprocessing and augmentation transforms to different medical imaging modalities such as CT or US, and improving compatibility with related works. The source code, as well as examples and documentation, are made publicly available online, on GitHub. We welcome feedback, feature requests, and contributions to the library, either by creating issues on the GitHub repository or by emailing the authors.

## Declaration of Competing Interest

The authors declare no conflicts of interest.

## Footnotes

1 https://github.com/PhoenixDL/rising.

2 https://brain-development.org/ixi-dataset/.

3 In this context, standardization refers to correcting voxel intensity values to have zero mean and unit variance.

4 https://github.com/fepegar/torchio.

5 https://zenodo.org/.

6 https://pypistats.org/packages/torchio.
